# Mucopolysaccharidosis Type I

**DOI:** 10.3390/diagnostics10030161

**Published:** 2020-03-16

**Authors:** Francyne Kubaski, Fabiano de Oliveira Poswar, Kristiane Michelin-Tirelli, Ursula da Silveira Matte, Dafne D. Horovitz, Anneliese Lopes Barth, Guilherme Baldo, Filippo Vairo, Roberto Giugliani

**Affiliations:** 1 Postgraduate Program in Genetics and Molecular Biology, UFRGS, Porto Alegre 91501970, Brazil; fkubaski@udel.edu (F.K.); fposwar@hcpa.edu.br (F.d.O.P.); umatte@hcpa.edu.br (U.d.S.M.); gbaldo@hcpa.edu.br (G.B.); 2 Medical Genetics Service, HCPA, Porto Alegre 90035903, Brazil; ktirelli@hcpa.edu.br; 3 INAGEMP, Porto Alegre 90035903, Brazil; 4 Biodiscovery Research Group, Experimental Research Center, HCPA, Porto Alegre 90035903, Brazil; 5 Gene Therapy Center, HCPA, Porto Alegre 90035903, Brazil; 6 Department of Genetics, UFRGS, Porto Alegre 91501970, Brazil; 7 Medical Genetics Department, National Institute of Women, Children, and Adolescent Health, Oswaldo Cruz Foundation, Rio de Janeiro 21040900, Brazil; dafne@ceresgenetica.com.br (D.D.H.); annebarth@hotmail.com (A.L.B.); 8 Department of Physiology, UFRGS, Porto Alegre 90050170, Brazil; 9 Center for Individualized Medicine, Mayo Clinic, Rochester, MN 55905, USA; Vairo.Filippo@mayo.edu; 10 Department of Clinical Genomics, Mayo Clinic, Rochester, MN 55905, USA; 11 Postgraduation Program in Medicine, Clinical Sciences, UFRGS, Porto Alegre 90035003, Brazil

**Keywords:** mucopolysaccharidosis type I, Hurler syndrome, Hurler–Scheie syndrome, Scheie syndrome, glycosaminoglycans, enzyme replacement therapy, hematopoietic stem cell transplantation

## Abstract

Mucopolysaccharidosis type I (MPS I) is caused by the deficiency of α-l-iduronidase, leading to the storage of dermatan and heparan sulfate. There is a broad phenotypical spectrum with the presence or absence of neurological impairment. The classical form is known as Hurler syndrome, the intermediate form as Hurler–Scheie, and the most attenuated form is known as Scheie syndrome. Phenotype seems to be largely influenced by genotype. Patients usually develop several somatic symptoms such as abdominal hernias, extensive dermal melanocytosis, thoracolumbar kyphosis odontoid dysplasia, arthropathy, coxa valga and genu valgum, coarse facial features, respiratory and cardiac impairment. The diagnosis is based on the quantification of α-l-iduronidase coupled with glycosaminoglycan analysis and gene sequencing. Guidelines for treatment recommend hematopoietic stem cell transplantation for young Hurler patients (usually at less than 30 months of age). Intravenous enzyme replacement is approved and is the standard of care for attenuated—Hurler–Scheie and Scheie—forms (without cognitive impairment) and for the late-diagnosed severe—Hurler—cases. Intrathecal enzyme replacement therapy is under evaluation, but it seems to be safe and effective. Other therapeutic approaches such as gene therapy, gene editing, stop codon read through, and therapy with small molecules are under development. Newborn screening is now allowing the early identification of MPS I patients, who can then be treated within their first days of life, potentially leading to a dramatic change in the disease’s progression. Supportive care is very important to improve quality of life and might include several surgeries throughout the life course.

## 1. Introduction

Despite being described by Dr. Gertrud Hurler in 1919, two years after the description of the first cases of mucopolysaccharidosis (MPS) by Dr. Charles Hunter, Hurler syndrome was classified as MPS I, along with other diseases that involve the storage of mucopolysaccharides (or glycosaminoglycans, GAGs) [1].

When cell complementation studies showed that MPS I and the former MPS V (Scheie disease, a milder form of MPS initially described in a patient with corneal clouding) shared the same basic defect [2], Scheie disease was considered to be the attenuated end of the MPS I spectrum, and the designation MPS V was removed to avoid confusion.

As some patients cannot be identified as having the severe Hurler phenotype (MPS IH) or as presenting the attenuated Scheie phenotype (MPS IS), an intermediate form called Hurler–Scheie syndrome (MPS IH-S) was proposed [3,4]. 

Nowadays, it is recognized that there is a severe phenotype that is relatively homogeneous and includes the patients with Hurler syndrome, and an attenuated phenotype that is quite variable and ranges from severe Hurler–Scheie cases to quite mild Scheie patients [5,6,7]. 

The clinical manifestations, laboratory diagnosis, and therapeutic approaches for MPS I are presented and discussed herein. Additionally, the current status of newborn screening and the genotype–phenotype correlations for this disease are also discussed.

## 2. Clinical Picture

### 2.1. Severe Phenotype (Hurler Syndrome)

The severe phenotype of mucopolysaccharidosis type I (MPS IH) is characterized by an early onset of somatic and neurological manifestations. 

Patients have global developmental delay that may progress to severe intellectual disability in those not treated with hematopoietic stem cell transplantation (HSCT). Furthermore, even in those who receive transplantation, there is usually a lower than average IQ and many patients still require special education [8]. 

Apart from the cognitive impairment, MPS IH patients have also severe somatic involvement. Many of those somatic manifestations may be identified in the first months of life, including abdominal hernias [6] and extensive dermal melanocytosis (Mongolian spots). The latter abnormality is also found in other neuronopathic lysosomal diseases, and may be the result of an abnormal melanocyte migration due to the interaction of the accumulated substrate and a receptor for the nerve growth factor (NGF) [9].

Thoracolumbar kyphosis (gibbus) is also one of the earliest manifestations of MPS IH, with a median age of onset of 1.0 years [6]. Other spinal abnormalities include spondylolisthesis and odontoid dysplasia. Progressive arthropathy may affect small and large joints, manifesting as joint restriction, camptodactyly of the fingers (claw hands), coxa valga, and genu valgum. The diaphyseal constriction in the proximal and medial phalanges may be absent, resulting in a bullet shape [10]. Foot and ankle abnormalities may include ankle valgus, camptodactyly of the toes, and planoabductovalgus feet [11]. As a result of the bone deformities, patients with severe MPS I typically have a very short final stature, although some may have overgrowth in early life [12].

Coarse facial features are among the most prominent manifestations of MPS IH [6]. The typical facial dysmorphism results form a combination of frontal bossing, broad nasal bridge, midface retrusion, and thickening of the lips. Hypertrichosis and oral abnormalities, including macroglossia, small and widely spaced teeth, and gingival overgrowth may also contribute to the gestalt [13,14]. Other described dental findings are open-bite, delayed eruption of the teeth, flattening or enlargement of the alveolar ridges, hypodontia, taurodontism, enamel defects, and abnormal morphology of the canine and molar crowns [14]. Abnormalities of the skull may include a J-shaped sella turcica, dolicocephaly, and thickening of the calvaria [13,15].

Recurrent upper and lower airway infections are very frequently described. Moreover, patients often have upper airway obstruction during sleep, resulting in moderate or severe obstructive sleep apnea [16]. In younger patients, chronic otitis media with effusion may result in conductive hearing loss that may be reversible with the insertion of ventilation tubes. In older patients, sensorineural hearing loss resulting from GAG accumulation in the cochlea, auditory nerve, and brainstem is also usually present [17].

Cardiovascular manifestations are also a prominent feature in MPS IH and may contribute to a shortened lifespan. Patients usually develop a progressive thickening of the cardiac valve leaflets, resulting in valvular insufficiency and/or stenosis, predominantly in the mitral and aortic valves [18]. Cardiovascular manifestations may also include left ventricle hypertrophy, systemic or pulmonary hypertension, coronary artery disease, and aortic root dilatation [19,20,21]. 

**Figure 1:**
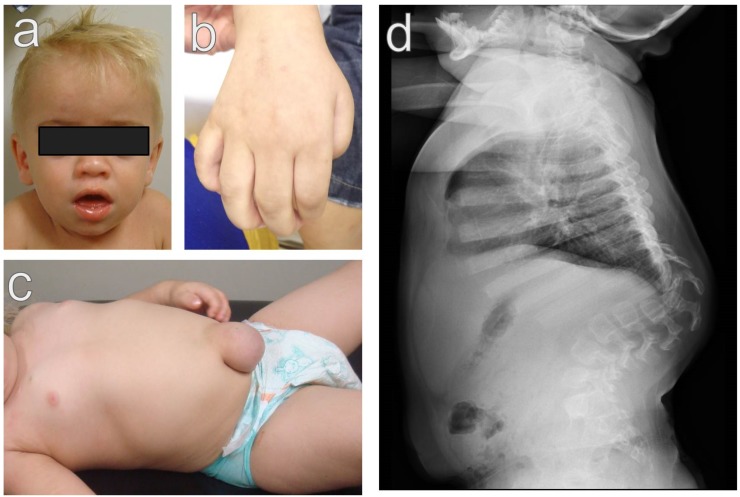
Hurler syndrome features. (**a**). Coarse facial features. Note the large forehead, short neck, broad nasal tip, coarse facial features, and corneal clouding. (**b**). Claw hands due to camptodactyly of the fingers. (**c**). umbilical hernia. (**d**). lateral view of the spine. Note the thoracolumbar kyphosis and the broad ribs. Informed consent was obtained for image use.

Glycosaminoglycan accumulation and disruption of the collagen fibrils in the corneal epithelium results in corneal opacity in the majority of the patients [22], resulting in low visual acuity that may be worsened by the presence of optic disc atrophy, glaucoma, retinopathy, and refraction errors, mainly hypermetropia, especially in older children with MPS IH [22]. Figure 1 shows some of the main manifestations of severe MPS I.

### 2.2. Attenuated Phenotypes

**Figure 2:**
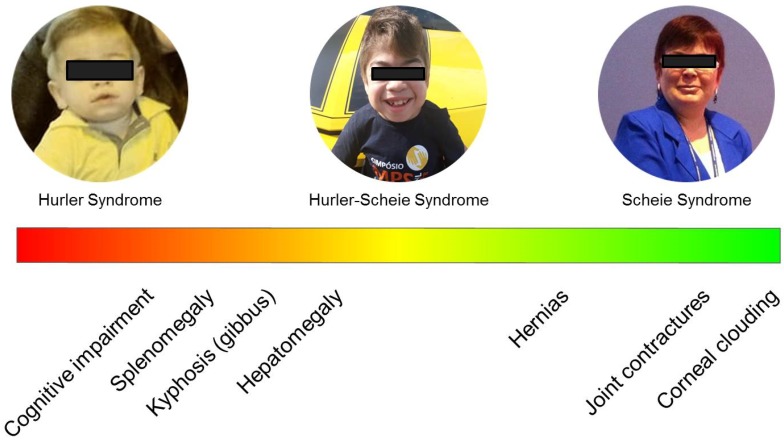
Spectrum of MPS I patients. Manifestations written to the left are more commonly found as presenting signs and symptoms in patients with the severe phenotypes, while those to the right are also frequently seen as presenting signs and symptoms in patients with attenuated phenotypes. Informed consent was obtained for image use.

Attenuated phenotypes are differentiated from the Hurler syndrome by the absence of developmental delay and the presence of a range of somatic manifestations that may be either more pronounced, in those with Hurler–Scheie syndrome (MPS IHS), or less pronounced, in those with Scheie syndrome (MPS IS). Nevertheless, it is now clear that there is a continuous spectrum of manifestations among those patients and a clear differentiation between the historical denominations of Hurler–Scheie and Scheie syndromes is not always possible (Figure 2). 

It is important to notice that a significant proportion of individuals with attenuated phenotypes of MPS I may have a later-onset cognitive decline. Indeed, data from a multicenter study revealed that, although individuals with an attenuated phenotype aged between 2 and 6 years had normal IQ scores, 43% of the patients in the age range of 6 to 25 years had either borderline or impaired cognitive ability [8]. Even in patients with no cognitive impairment, white matter abnormalities are usually present [23] and may occasionally be diffuse, mimicking a leukodystrophy [24]. 

The somatic manifestations may include most of those mentioned previously, although they are usually reduced in number and less severe. As an example, in the milder cases, there may not be any significant oral abnormality, except those restricted to the mandible, including a short mandibular ramus with a flat mandibular notch and condylar defects [25]. Conversely, some of the manifestations may be more frequently described in patients with MPS IS as compared to patients with MPS IH, including joint contractures and carpal tunnel syndrome [6], probably due to the greater lifespan of patients with attenuated phenotypes.

## 3. Laboratory Diagnosis

### 3.1. Glycosaminoglycans

Glycosaminoglycans (GAGs) are long, negatively charged polysaccharides that are essential for several cellular processes. In the absence of the lysosomal hydrolase alfa-l-iduronidase (IDUA), two major subclasses of GAGs (dermatan sulfate, DS; and heparan sulfate, HS) are accumulated inside the lysosomes of a wide range of tissues [26]. Thus, GAG analysis is highly useful for the diagnosis and therapeutic monitoring of MPS [27,28,29].

GAG analysis has been used for the diagnosis of MPS since the early 50s [30,31,32,33,34,35,36]. One of the most widely used methodologies for the analysis of GAGs is the use of dimethylmethylene blue (DMB), a cationic dye that binds to the sulfated GAGs and allows measurement based on its absorbance, with reference ranges that are age-dependent [37,38,39]. Careful examination should be conducted for the presence of heparin, artificial coloring agents, and the amount of protein in the sample [37,40,41]. Nevertheless, this method is still used for the screening of MPS patients, with reproducible results and low cost, while it still has the disadvantages of not applying to blood and tissue samples directly, and it also does not discriminate between specific GAG subclasses [26].

It is very useful to quantify the levels of GAGs accumulated, as well as to know which specific subclass is being stored. Thus, the use of tandem mass spectrometry (MS/MS) to quantify GAGs has been explored in several matrices such as cell lines [42], urine [34,35,43], tissue extracts [36], plasma/serum and or urine [44,45,46], dried blood spots [27,28], and cerebrospinal fluid [47]. Besides the different biological matrices, several approaches exist for GAG quantification including analysis of disaccharides [28,34], chemical degradation by methanolysis [43,47], and non-reducing ends [48,49].

Regardless of the method used, GAG analysis is a very useful approach for the diagnosis, treatment follow-up, and prognosis of the patients [27,28,45,50]. With the advent of NBS for MPS, GAGs in DBS are essential for the quick identification of false positives [51,52].

### 3.2. Enzyme Assay

The gold standard for the diagnosis of MPS I is based on measurement of the residual activity of alfa-l-iduronidase (IDUA), which can be measured by fluorimetry with 4-methylumbelliferyl (4-MU) as a substrate [53,54]. The enzyme assay is performed in acidic pH in plasma, leukocytes, fibroblasts, amniocytes, or chorionic villi (direct assay or in cultured cells). The substrate 4-MU is an artificial fluorogenic substrate that, when degraded by IDUA, releases methylumbelliferone as a fluorescent fraction that can be measured using a calibration curve. The enzyme activity is usually expressed in nmoL/h/mg of protein or as nmoL/h/mL (plasma). IDUA can also be assayed by high-throughput fluorimetry with a digital microfluidics (DMF) system. DMF platforms consist of electrowetting reactions in which liquids are processed as individual droplets that are mixed, separated, and then transported onto the cartridge [55,56,57]. The major benefits of DMF are the simplicity, the smaller amounts of volume for samples and reagents, and the shorter incubation allowing large-scale testing with a low cost [56].

Nowadays, the most robust measurement technique for IDUA is tandem mass spectrometry (MS/MS), in which IDUA can be assayed from rehydrated dried blood spots (DBS) [52,58]. This assay is currently used in the NBS of MPS I (see NBS section for more details) [59,60].

**Figure 3:**
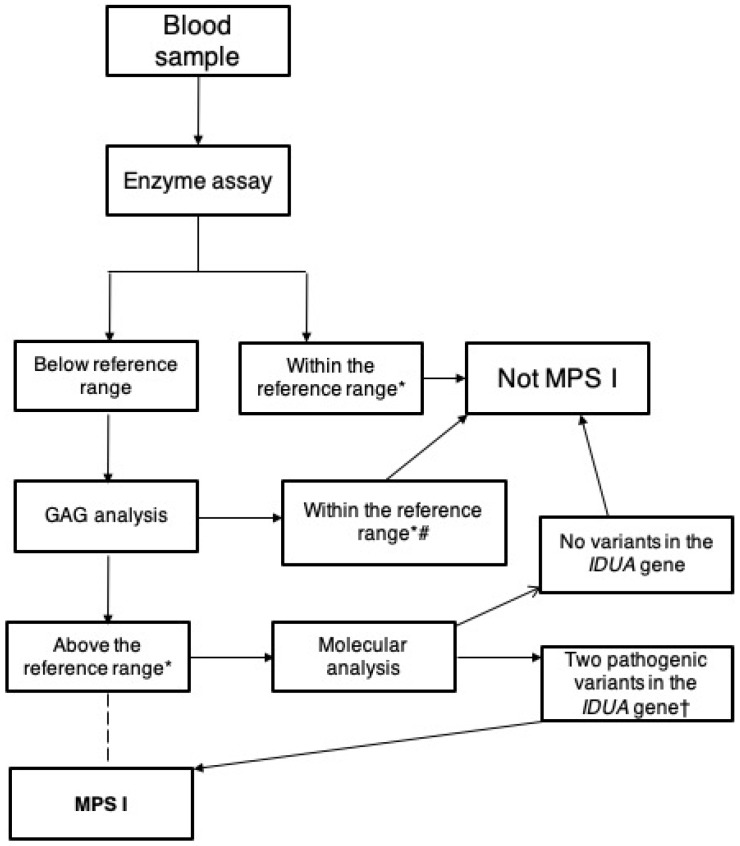
Flowchart for the newborn screening of MPS I (this flowchart could be used to investigate symptomatic subjects, although in these cases the investigation usually starts with the quantitative and/or qualitative evaluation of urinary GAGs, positive cases being referred for the blood testing). * Reference ranges for alfa-l-iduronidase (IDUA) and glycosaminoglycans may vary according to the methodology used and must be established in each laboratory. # Pseudodeficiency leading to low IDUA levels with normal GAG levels. † Careful examination should be performed with variants of unknown significance (VUS). If no variant is found with Sanger or next-generation sequencing, other techniques such as multiplex ligation-dependent probe (MLPA) should be performed. It is also always recommend to analyze the trio (proband and parents). The dashed line for MPS I diagnosis represents the choice of some laboratories to report a diagnosis solely based on enzyme levels and GAG analysis without molecular analysis of the *IDUA* gene; this approach has been used in NBS at some centers.

Careful analysis should be performed with samples that show low IDUA activity in vitro with no altered GAG metabolism; this is caused by the presence of pseudodeficiency alleles [61]. These alleles are present in different frequencies across different ethnicities. It is also important to mention that no biochemical correlation exists for enzyme quantification to predict disease phenotypes [62]. Figure 3 shows a flowchart of the diagnosis of MPS I based on enzyme assay, GAG analysis, and molecular analysis.

### 3.3. Molecular Diagnosis

Molecular diagnosis of MPS I is traditionally viewed as a confirmatory test after biochemical diagnosis (Figure 3). It is quite important, though, in cases with unclear enzyme results and particularly for prenatal diagnosis in affected families. More recently, molecular diagnosis has been recognized as an important part of the diagnostic process, especially since therapeutic options have become available and genotype–phenotype correlations have become clearer [62,63]. 

Molecular analysis of MPS I patients is usually performed by complete sequencing of the *IDUA* gene using next-generation sequencing (NGS) or Sanger sequencing of each one of the 14 exons. NGS can be performed as part of whole-genome or exome sequencing or as part of a targeted gene panel. The latter approach is more cost-effective, especially in centers dedicated to lysosomal storage disorders. In our experience, caution must be taken when performing NGS for *IDUA* due to its high G–C content, which may result in lower coverage, especially for Exons 1 (78% G–C content), 9 (77%), and 10 (74%). However, validation of a gene-targeted panel for MPS showed high sensitivity and specificity [64]. Variant prioritization should be performed by someone with experience in MPS I.

Over 300 variants in the *IDUA* have been reported in the Human Genetic Mutation Database (HGMD) [65], with frequencies that differ across populations [66]. Table 1 shows the most frequent pathogenic variants and their combined frequency. Importantly, 3% of variants are gross deletions, insertions, or complex rearrangements that would be missed by traditional sequencing approaches such as Sanger sequencing or NGS-based techniques. Therefore, in confirmed cases with only one pathogenic variant detected, alternative techniques such as gene dosage or MLPA should be applied. Rare cases of maternal mosaicism have been described [67] and at least two pseudodeficiency alleles are known: p.His82Gln [68] and p.Ala300Thr [61]. Therefore, variant interpretation must be performed with caution, and preferably in conjunction with biochemical and clinical data.

## 4. Therapeutic Approaches

### 4.1. Hematopoietic Stem Cell Transplantation

The first hematopoietic stem cell transplantation (HSCT) for MPS was performed in 1980 [69], with the rationale of providing enzyme activity to metabolically deficient individuals [70]. Several mechanisms could contribute to a beneficial effect: first, the replacement of enzyme-deficient cells by normal cells is important, especially for diseases in which the mononuclear phagocytic cell system is mainly affected. After HSCT, the donor’s enzymatically competent macrophages gradually replace the recipient’s macrophages, which are heavily loaded with non-metabolized storage material. Second, the enzyme is transferred from the enzymatically normal donor-derived cells to deficient cells by direct cell–cell contact. HSCT may also be effective via the release of enzyme into plasma, for example by disintegration of donor-derived white blood cells. The circulating enzyme may be taken up by enzymatically deficient cells. The final important mechanism may be a concentration gradient of storage product between the tissues and the plasma compartment, which may result from the breakdown of circulating substrate by the lysosomal enzyme in white blood cells and tissue macrophages of donor origin. Such a gradient may lead to clearance of storage product [71]. 

The term HSCT includes the use of umbilical cord blood cells (UCB) or bone marrow as grafts. It is considered the gold standard treatment for the severe type of MPS I (Hurler), ideally being undertaken as early as possible—before 2.5 years of age and before severe cognitive impairment, since it may take around a year for donor-derived cells to reach a significant presence in the brain and replace the existing microglial cells [72,73,74]. HSCT is expected to improve the clinical manifestations of MPS, including reduced joint mobility, vision, hearing, cardiopulmonary function, coarsened facial features, upper airway obstruction, respiratory function, and hepatosplenomegaly. The transplant can also stabilize or prevent hydrocephalus and prevent the deterioration of psychomotor functions. It is important to point out, however, that this treatment strategy is not able to significantly correct clinical manifestations of the disease in bone or cornea, cardiac valve abnormalities, or preexisting cognitive and intellectual effects [75]. The access to CNS and correction of metabolism in nervous tissue is the main reason for HSCT being considered the treatment of choice for the severe form of MPS I (Hurler), since intravenous enzyme replacement therapy (ERT) does not cross the blood–brain barrier. However, it should be performed early to achieve this goal and to justify the risks of HSCT, which, although they have been reduced since its development, are not negligible [5,76].

The effectiveness of HSCT depends on the level of enzyme activity achieved after transplantation, which is directly related to the type of donor cell (cord blood versus bone marrow, non-carrier donor) and chimerism [75,77,78,79]. To date, more than 1000 MPS patients have received HSCT as a treatment option for their disease [75]. Experienced HSCT centers have reported engrafted survival rates of 90% for MPS I patients, primarily due to the updated guidelines from the European Society for Blood and Marrow Transplantation (EBMT) protocol and selection of well-suited donors [75,80].

For the milder/later phenotypes of MPS I (Hurler–Scheie and Scheie), due to their slower progression, longer life expectancy, and lower or absent cognitive impairment, there is not a consensus supporting the use of HSCT, especially since the availability of ERT [74]. Nevertheless, such a strategy has been used in several countries and is being revised, considering the high cost of lifelong ERT versus one-time HSCT treatment. With new conditioning protocols, transplantation has become safer [81], although complication risks still exist.

### 4.2. Intravenous Enzyme Replacement Therapy

Enzyme replacement therapy is part of the standard of care for patients with MPS I. It may be used either as a monotherapy or as an adjuvant therapy prior to and during transplantation of patients with severe MPS I. Currently, laronidase is the only commercially available product. It received FDA approval after the results of a double-blind phase III clinical trial [82] (Table 2). A recently published Cochrane review, which included only that study, pointed out that the evidence is sufficient to demonstrate that laronidase is effective in improving biochemical and functional parameters, although more studies are necessary to assess its long-term efficacy [83].

Evidence for long-term efficacy has been provided by open-label studies. Interpretation of the results from open-label observational studies with laronidase may be difficult due to the high prevalence of concomitant therapies, which may also influence in the outcomes [84]. In spite of that, data from those studies suggest that long-term treatment with laronidase may result in sustained improvement, but not normalization, of plasma and urinary biomarkers [85,86], and improvements in many manifestations including the liver volume [87], shoulder range of motion [87], and pulmonary function tests [87,88]. Nevertheless, the effects on the heart valve disease, corneal clouding, and skeletal manifestations are not consistent [87,89,90].

The production of antibodies to laronidase occurs in almost all patients and high titers, per se, do not predict a lower clinical response [82]. However, the presence of inhibitory antibodies, as assessed by the combination of serum antibody titers towards ERT and a cellular uptake inhibition assay, has been shown to correlate with higher dermatan sulfate:chondroitin sulfate (DS:CS) ratios and to be associated with the severity of the sleep-disordered breathing [88]. 

A major limitation of intravenous ERT is its inability to cross the blood–brain barrier in significant amounts. Although treatment with ERT was shown to improve white matter abnormalities and perivascular spaces in patients with Hurler and Hurler–Scheie syndromes [91], ERT is not sufficient to prevent neurodegeneration in MPS I patients, which stresses the need for new therapies. 

### 4.3. Intravenous Enzyme Replacement Therapy with Fusion Proteins

A blood-brain barrier (BBB)-penetrating form of IDUA would allow a non-invasive therapeutic option for treatment of the central nervous system (CNS) manifestations in MPS I. One approach used was to make the IDUA enzyme transportable through the BBB following the re-engineering of the lysosomal enzyme as an IgG–IDUA fusion protein, where the IgG domain is a receptor-specific monoclonal antibody (MAb) that targets an endogenous BBB receptor transporter.

The IgG–IDUA fusion protein was formed by fusion of the human IDUA enzyme, without the enzyme signal peptide, to the carboxyl terminus of each heavy chain of a genetically engineered chimeric HIRMAb, that binds the human insulin receptor (HIR) [92]. The HIRMAb domain of the HIRMAb–IDUA fusion protein triggers receptor-mediated transport of the fusion protein into the brain via the endogenous BBB insulin receptor, and acts as a molecular Trojan horse to ferry into the brain the IDUA fused to the IgG domain [92,93]. The HIRMAb–IDUA fusion protein is designated as valanafusp alpha. 

A surrogate fusion protein reduced lysosomal inclusion bodies in the brain of an MPS I mouse model following chronic IV administration [94]. The IDUA domain of valanafusp alpha incorporates mannose 6-phosphate (M6P) [93], which allows uptake also into somatic tissues via the M6P receptor (M6PR), similar to recombinant IDUA. Whole body autoradiography in primates showed a comparable biodistribution in peripheral organs for laronidase and valanafusp alpha [93]. However, the M6PR is not expressed at the human BBB, and laronidase does not penetrate the monkey brain [93]. Conversely, there was global penetration of the CNS by valanafusp alpha in the primate model owing to BBB transport of the fusion protein via the endogenous insulin receptor [93]. The dual-receptor targeting of the HIRMAb–IDUA fusion protein provided the rationale for reversal of lysosomal inclusions in both somatic and CNS tissues following chronic IV treatment of MPS I subjects with valanafusp alpha. 

A phase I–II clinical trial of the treatment of MPS I adults and children with valanafusp alpha was reported. After a single dose-escalation phase I trial in six adult MPS I subjects, a phase II trial in 11 pediatric MPS I patients (Hurler and Hurler–Scheie phenotypes) was performed over 52 weeks of chronic weekly IV infusions of valanafusp alpha at doses of 1, 3, and 6 mg/kg. The authors concluded that valanafusp alpha presented a favorable safety profile and that weekly IV infusions of valanafusp alpha stabilized CNS function and somatic manifestations of the disease [95]. These results provided support for the further development of this drug. 

Another approach is the use of fusion proteins that, instead of binding to the insulin receptor, bind to the transferrin receptor. This strategy has already been submitted to a phase I/II clinical trial for patients with MPS II [96], and is in preclinical development for MPS I. 

### 4.4. Intrathecal Enzyme Replacement Therapy

Intrathecal administration of ERT is being assessed as an option for the treatment of central nervous system manifestations of MPS I, including spinal cord compression and cognitive decline. In the first published case report, a 38 year old male with spinal cord compression received four monthly intrathecal injections. The administration was uneventful, except for a minor bleeding in the fourth dose. After the treatment, the disappearance of a right ankle clonus and an improvement in temperature sensation was noted [97]. More recently, a phase I clinical trial of intrathecal laronidase also evaluated its use in subjects with cervical stenosis [98]. Laronidase was diluted in Elliot’s B and administered every 30 days for 4 months. A total of five patients were recruited, all of whom had symptomatic spinal cord compression that did not require urgent surgical intervention. Three subjects completed the pilot study and reported subjective improvements in the symptoms of spinal cord compression. Only one subject completed a 1 year extension study. The results of the study suggested that intrathecal laronidase is safe and well-tolerated, but data were insufficient for efficacy analysis.

Another study assessed the impact of intrathecal laronidase for cognitive decline in a 23 year old male with MPS IHS [99]. The patient received monthly doses for 3 months, and then doses every 3 months for 24 months. He had improvements in mean IQ scores of all subtests and reduction of hippocampal volumes, suggesting a promising role of intrathecal ERT for MPS-I-related cognitive decline.

### 4.5. Gene Therapy, Gene Editing, and Stop Codon Read Through

The fact that IDUA is secreted and HSCT can prevent cognitive decline suggests that both in vivo and ex vivo gene therapy approaches could work in MPS I. In vivo gene therapy implies indirect injection of the vector either into the blood or in situ. In this scenario, initially, retroviruses showed good results in both mouse and dog models [100], but the risk of insertional oncogenesis prevented their proceeding to clinical trials. In recent years, different AAV serotypes have shown promising results [101], and gene therapy clinical trials for MPS I using these vectors are now becoming a reality (as an example: NCT03580083 with AAV now recruiting)

Gene editing using zinc-finger nucleases (ZFN) aiming at adding a copy of *IDUA* at the albumin locus improved several parameters in the mouse model [102], but initial clinical results demonstrated that, despite being safe, the increase in serum IDUA levels was too small. Similar results were found in MPS II patients using the same approach [103]. These results were received with some disappointment, although protocol adjustments are expected to potentially modify the initial findings. Ex vivo gene editing in Hurler mice was recently described. In this study, it was possible to modify the genome of human HSCs at the CCR5 locus, inserting a copy of the *IDUA* at this position. The cells were transplanted into MPS I mice, and engraftment of cells resulted in their biodistribution to most organs, with reduction in tissue GAGs and improvement in behavioral parameters, suggesting the prevention of brain disease [104]. Off-target effects were studied, with no signs of genotoxicity. This could allow autologous transplantation of modified cells, which would avoid several problems associated with traditional HSCT.

Most MPS I patients in Brazil, USA, Australia, and some European countries carry a nonsense mutation (p.Trp402Ter), which makes the stop codon read through (SCRT) strategy a potential treatment option [66]. SCRT consists of treating the patient with a molecule that can interact with the ribosome and add an amino acid into the protein instead of the premature stop codon, meaning that the protein can be produced. In MPS I, different molecules have been tested in patients’ cells and in animal models, including lividomycin and NB54 [105,106]; however, the efficacy of the system seems to be too low to produce a clinical benefit. Aminoglycosides such as gentamicin and chloramphenicol have been tested and showed promising results in mice and patient cells [106,107]. PTC124 (Ataluren™) is a non-aminoglycoside compound that can read through nonsense variants and has been evaluated in a phase II trial in patients with MPS I (EudraCT Number:2014-002596-28). However, no results have been reported to date.

### 4.6. Small Molecules

Small molecules are substances that have the potential to alter GAG degradation or synthesis and can cross the BBB, so are possible options to treat the CNS involvement in the neuronopathic forms of MPS. Rhodamin B is a non-specific inhibitor of GAG synthesis and has been proven to reduce GAG excretion and improve neurological and somatic findings in mice with MPS I. Since inflammation may be involved in the pathophysiology of some MPS I features, trials with anti-inflammatory therapies such as TNF-alpha-antagonists and pentosan polysulfate (PPS) have been attempted. For example, adalimumab showed some benefits for pain and physical function in children with MPS I [108] while PPS reduced urinary GAG excretion and improved joint mobility and pain in adult MPS I individuals [109]. 

## 5. Newborn Screening

Newborn screening (NBS) focuses on the early identification of disorders to allow early intervention avoiding irreversible life-threatening manifestations [110]. It is very clear that MPS I patients have great benefit from early treatment, justifying its inclusion in the NBS programs [1,52,60,62,111,112]. 

The US Department of Health and Human Services recommended the addition of MPS I to the recommended uniform screening panel (RUSP) in 2016 (https://www.hrsa.gov/sites/default/files/hrsa/advisory-committees/heritable disorders/rusp/previous-nominations/mps-i-27-june-2018.pdf Accessed: 28 October 2019). 

**Figure 4:**
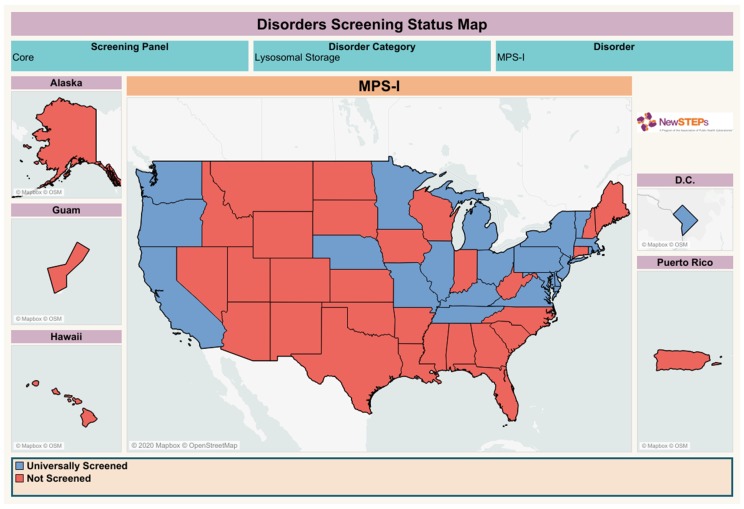
Status of the MPS I NBS in the United States. Current status available from the NewSTEPs (https://www.newsteps.org/resources/newborn-screening-status-all-disorders, Accessed: 14 March 2020).

Currently, 21 states from the United States are universally screening for MPS I (Figure 4). Five states are performing the assay by DMF, 11 by MS/MS, and information about the methodology for the other five states was not available at the NewSTEPs. Ten states are currently pursuing implementation/seeking authorization and/or funding (Utah, New Mexico, South Dakota, Iowa, Florida, South Carolina, North Carolina, Indiana, Connecticut, and Hawaii).

Some other countries that have conducted pilot studies for MPS I include Taiwan [60,113], Italy [51,114,115], Austria [116], Brazil [117], Belgium [118], and Mexico [119].

Another approach that has been used for the screening of MPS I is the use of GAG quantification as a first-tier assessment based on the quantification of dermatan sulfate (DS) and heparan sulfate (HS) followed by quantification of IDUA as a second-tier strategy. The pilot study showed a high level of false positives (0.03%) [28]. This false-positive rate is too high, compared to <0.008% for IDUA quantification by MS/MS [120]. Thus, the GAG assay should be performed as a second-tier strategy to discriminate affected patients from pseudodeficiency [121].

Currently, the major limitations associated with MPS I NBS are due to the lack of clear correlations of biochemical diagnosis (enzyme and GAG levels) to predict subtypes and unclear genotype–phenotype correlations with several private variants in the *IDUA* gene, higher rates of pseudodeficiency with limited information about pseudodeficiency genotypes, and variants of unknown significance (VUS) [62,122]. 

NBS programs might vary the algorithms used for first-tier testing (DMF or MS/MS) and second-tier testing (GAG analysis and/or *IDUA* sequencing), but it is clear that regardless of the chosen methodology, affected infants will benefit from early intervention.

## 6. Genotype–Phenotype Correlation

Historically, MPS I individuals were classified into three categories of increasing severity, Hurler, Hurler–Scheie, and Scheie, with the intermediate form thought to be caused by compound heterozygous variants [123]. Nowadays, it is known that the phenotype is determined by the allele with the highest residual enzymatic activity. Although recognized as a continuum of severity, the categorization of MPS I individuals into attenuated or severe forms is helpful for clinical management and treatment decisions. Several countries have included MPS I in their newborn screening panels, so many individuals have been identified when the neurological symptoms are inexistent or minimal. 

Unfortunately, enzymatic testing and GAG analysis cannot predict whether a patient will have a severe or attenuated phenotype. On account of this, best practice guidelines suggest the use of *IDUA* genotype in the decision-making process [62]. 

Over 300 variants in *IDUA* have been reported in the Human Gene Mutation Database (HGMD) 65]. Two common nonsense variants, p.Trp402* and p.Gln70*, together account for 63% of the causative variants for MPS I in Caucasians [66]. Individuals who are homozygous or compound heterozygotes for these variants have severe phenotype. In 2003, Sanofi-Genzyme (Cambridge, Massachusetts, USA) launched the MPS I Registry (https://clinicaltrials.gov, NCT00144794) to gather retrospective and prospective clinical, biochemical, and genotype information from over 530 individuals with MPS I, which aided in delineating a better genotype–phenotype correlation [63]. 

Almost 80% of individuals with severe phenotypes have loss-of-function variants, whereas only 23% of individuals with attenuated disease harbor this type of variant. The p.[Trp402*];[Trp402*], p.[Trp402*];[Gln70*], and p.[Gln70*];[Gln70*] genotypes account for 51% of severe patients’ genotypes. The most common genotypes in attenuated patients are p.[Leu490Pro];[Leu490Pro], p.[Pro533Arg];[Pro533Arg], and p.[Leu238Gln];[Trp402*], which are present in 28% of individuals with attenuated phenotypes. 

Homozygosity or compound heterozygosity for two severe variants always leads to severe disease. Even though 97.5% of individuals with attenuated phenotypes harbor at least one missense variant and over 45% are heterozygous for a loss-of-function variant, several genotypes have been associated with both types of disease. For instance, p.[Pro533Arg];[Pro533Arg] has been seen in seven individuals with severe phenotypes and 17 individuals with the attenuated form. Data from the MPS I Registry show that over 50% of the affected individuals have unique genotypes and several missense variants are associated with multiple phenotypes, making it impossible to predict the phenotype solely based on the genotype information for a vast number of patients [63]. Table 3 describes a summary of genotypes and the number of patients within each of the disease forms reported in the MPS I Registry.

## 7. Prospects and Conclusions

One century after the report of the first cases of MPS I, many advances in the understanding of this complex disease have been achieved. Several tools have also been developed for its diagnosis, including biochemical and genetic techniques that enable patient diagnosis, carrier identification, and prenatal diagnosis. 

The first successful therapeutic intervention (bone marrow transplantation) was reported in 1980, and in 2003, intravenous replacement therapy with a recombinant enzyme (laronidase) became available. 

Although these therapeutic approaches transform the natural history of the disease, several problems remain, with unmet needs related to the CNS manifestations of the disease, the skeletal abnormalities, the heart valve problems, and the corneal clouding, among others. This has stimulated investigators to develop new therapeutic approaches, which now include the delivery of enzymes directly to the CNS, the use of fusion proteins that bypass the BBB, small molecules that improve specific manifestations, strategies that improve translation of the protein, gene therapy, and gene editing. Even if these therapies are successful, a major challenge will remain in the identification of patients early enough, before irreversible manifestations occur. This has been a powerful argument for the promotion of newborn screening for MPSI, which is now a reality in several countries and regions. The combination of effective therapies and early diagnosis should dramatically change the prospects of MPS I patients in the not-too-distant future. 

## Figures and Tables

**Table 1:** Most common variants in MPS I patients worldwide (modified from Reference [66].

Variant ^a^	Combined Frequency (%)
p.Trp402 *	31.5
p.Pro533Arg	25.8
p.Gln70 *	21.5
c.56_47del12	6.3
p.Arg89Gln	6.3
p.Arg628 *	5.5
p.Gln380Arg	5.1
p.Ala327Pro	4.9
p.Glu178Lys	3.4

^a^ Only includes variants described in at least five different countries. Note that variants frequent only in Asian countries (such as c.1190-1 g>a and p.Leu346Arg) are not represented in this table. * nonsense variant.

**Table 2:** Therapeutic approaches approved and in development for MPS I.

Therapy	Category	Delivery	Current Status of Development	ClinicalTrials.Gov Identifier
**Laronidase (Aldurazyme^®^)**	Enzyme replacement therapy	I.V. infusion	Approved	NA
**Stem cell transplantation**	Stem cell transplantation	I.V. infusion	Approved	NA
**Intrathecal Laronidase**	Intrathecal Enzyme replacement therapy	Intrathecal administration	Phase I	NCT00638547, NCT00852358
**SB-318**	Genome editing	I.V. infusion	Phase I/II	NCT02702115
**RGX-111**	Gene therapy	Intracisternal injection	Phase I	NCT03580083
**AGT-181**	Enzyme replacement therapy with fusion protein	I.V. infusion	Phase I/II	NCT02597114, NCT03071341, NCT02371226, NCT03053089
**Genetically modified autologous hematopoietic stem cells**	Ex vivo gene therapy	I.V. infusion	Phase I/II	NCT03488394

I.V.: intravenous; NA: Not applicable.

**Table 3:** Genotype–phenotype correlation in MPS I.

Genotype	Severe Phenotype (*n* = 380)	Attenuated Phenotype (*n* = 158)
c.1403-1G> T;p.[Gln400*]	2 (0.5%)	0
c.1524+1G>T;p.[Trp402*]	2 (0.5%)	0
c.1650+5G>A;p.[Trp402*]	3 (0.8%)	0
c.1727+5G>C;p.[Asn348Lys]	0	2 (1.3%)
c.386-2A>G;c.386-2A>G	2 (0.5%)	0
c.386-2A>G;p.[Trp402*]	2 (0.5%)	0
p.[Ala327Pro];[Ala327Pro]	3 (0.8%)	2 (1.3%)
p.[Ala327Pro];[Arg383His]	0	1 (0.6%)
p.[Ala327Pro];[Arg89Trp]	0	1 (0.6%)
p.[Ala327Pro];[Gln380Arg]	0	1 (0.6%)
p.[Ala327Pro];[Gln70*]	5 (1.3%)	0
p.[Ala327Pro];[Ser423Arg]	1 (0.2%)	0
p.[Ala327Pro];[Thr374Asn]	0	1 (0.6%)
p.[Ala327Pro];c.1190-1G>C	1 (0.2%)	0
p.[Ala327Pro];[Ala327Pro]	0	2 (1.3%)
p.[Ala327Pro];[Trp402*]	14 (3.7%)	0
p.[Ala36Glu];[Gln70*]	0	2 (1.3%)
p.[Ala75Thr];[Gln70*]	2 (0.5%)	0
p.[Ala75Thr];[Trp402*]	2 (0.5%)	0
p.[Arg383His]; c.386-2A>G	0	3 (1.9%)
p.[Arg383His];[Gln70*]	0	2 (1.3%)
p.[Arg383His];[Trp402*]	0	2 (1.3%)
p.[Arg619*];[Trp402*]	5 (1.3%)	0
p.[Arg628*];[Arg628*]	6 (1.6%)	0
p.[Arg89Gln];[Trp402*]	0	4 (2.5%)
p.[Arg89Trp];[Trp402*]	0	3 (1.9%)
p.[Asn110Asp];[Gln70*]	2 (0.5%)	0
p.[Asn348Lys];[Trp402*]	0	2 (1.3%)
p.[Gln380*];[Arg654*]	0	2 (1.3%)
p.[Gln380Arg];[Gln380Arg]	0	4 (2.5%)
p.[Gln380Arg];[Thr388Arg]	0	2 (1.3%)
p.[Gln380Arg];[Trp402*]	0	2 (1.3%)
p.[Gln70*];[Gln70*]	24 (6.3%)	0
p.[Gly265Arg];[Trp402*]	0	2 (1.3%)
p.[Gly51Asp];[Gly51Asp]	2 (0.5%)	0
p.[Leu18Pro];[Thr388Lys]	2 (0.5%)	0
p.[Leu218P];[Gln70*]	5 (1.3%)	0
p.[Leu218P];[Leu218P]	2 (0.5%)	0
p.[Leu218P];[Trp402*]	3 (0.8%)	0
p.[Leu238Gln];[Gln70*]	0	2 (1.3%)
p.[Leu238Gln];[Trp402*]	0	6 (3.8%)
p.[Leu490Pro];[Leu490Pro]	0	21 (13.3%)
p.[Leu535Phe];[Trp402*]	0	2 (1.3%)
p.[Lys153*];[Trp402*]	2 (0.5%)	0
p.[Pro21fs];[Trp402*]	3 (0.8%)	0
p.[Pro496Arg];[Gln70*]	4 (1.1%)	0
p.[Pro533Arg];[Arg363Leu]	2 (0.5%)	0
p.[Pro533Arg];[Arg621*]	0	1 (0.6%)
p.[Pro533Arg];[Arg89Gln]	0	1 (0.6%)
p.[Pro533Arg];[Asp184Val]	0	1 (0.6%)
p.[Pro533Arg];[Asp301Glu]	2 (0.5%)	0
p.[Pro533Arg];[Gln70*]	0	2 (1.3%)
p.[Pro533Arg];[Gly197Asp]	0	1 (0.6%)
p.[Pro533Arg];[His425Profs*84]	1 (0.2%)	0
p.[Pro533Arg];[IleI259Asn]	0	1 (0.6%)
p.[Pro533Arg];[Lys153*]	0	1 (0.6%)
p.[Pro533Arg];[Pro533Arg]	7 (1.8%)	17 (10.8%)
p.[Pro533Arg];[Ser633Leu]	0	1 (0.6%)
p.[Pro533Arg];[Trp402*]	5 (1.3%)	3 (1.9%)
p.[Pro533Arg];[Trp402*]	0	3 (1.9%)
p.[Ser16del];[Glu178Lys]	0	2 (1.3%)
p.[Ser16del];[Trp402*]	6 (1.6%)	0
p.[Ser633Leu];[Trp402*]	0	3 (1.9%)
p.[Ser633Leu];[S16_A19del]	0	3 (1.9%)
p.[Thr388Arg];[Trp402*]	5 (1.3%)	0
p.[Trp402*];[Gln70*]	61 (16.1%)	0
p.[Trp402*];[Trp402*]	109 (28.7%)	0
